# Effects of artichoke extracts and probiotic on maternal immunological activation, valproic acid-induced oxidative stress, and gut leakiness in a rat model of autism

**DOI:** 10.1515/biol-2025-1355

**Published:** 2026-08-18

**Authors:** Maha K. Alaskar, Mona Alonazi, Abir Ben Bacha, Abdulaziz Mohammad Alamri, Musarat Amina, Nawal M. Al Musayeib, Sameera Abuaish, Afaf K. El-Ansary

**Affiliations:** Department of Biochemistry, College of Sciences, King Saud University, Riyadh 11495, Saudi Arabia; Department of Chemistry, College of Science, Imam Mohammad Ibn Saud Islamic University (IMSIU), Riyadh 11623 Saudi Arabia; Department of Pharmacognosy, College of Pharmacy, King Saud University, Riyadh 11495, Saudi Arabia; Department of Basic Sciences, College of Medicine, Princess Nourah Bint Abdulrahman University, Riyadh 11671, Saudi Arabia; Autism Research and Treatment Center, Riyadh 11495, Saudi Arabia

**Keywords:** autism spectrum disorder, valproic acid, maternal immune activation, lipopolysaccharide, artichoke, probiotic

## Abstract

Maternal health during pregnancy is a leading factor influencing offspring risk. This study investigated whether postnatal dietary supplementation could reduce oxidative stress and gut leakiness in a rat model of autism spectrum disorder (ASD). Male rat pups were prenatally exposed to valproic acid (VPA) or lipopolysaccharide (LPS) to induce ASD-like conditions. A total of 54 offspring of Wistar albino rats were divided into nine groups to evaluate various postnatal treatments, including an artichoke-based prebiotic (AR), probiotics (Pro), and omega-3 fatty acids (ω3). The experimental design also included control groups (saline, VPA-only, and LPS-only), as well as a protective regimen in which AR was administered both prenatally and postnatally. Oxidative stress and gut permeability “leakiness” were assessed using Enzyme-Linked Immunosorbent Assay (ELISA). Prenatal exposure to VPA and LPS was associated with increased oxidative stress levels in brain homogenates, accompanied by a significant decrease in glutathione (GSH). Additionally, elevated plasma levels of gut permeability biomarkers were observed. In the VPA model, treatment with artichoke-derived prebiotics – administered either prenatally, postnatally, or in combination with probiotics – effectively improved oxidative stress markers, as evidenced by a significant increase in GSH levels. Conversely, similar interventions in the LPS-induced maternal immune activation model did not significantly ameliorate oxidative stress, although a modest increase in GSH levels was noted. Plasma levels of gut permeability biomarkers did not show significant improvement in either model following treatment with artichoke-derived probiotics alone or in combination with probiotics and omega-3 fatty acids. However, intestinal fatty acid-binding protein levels were significantly reduced in all treatment groups across both models. In contrast, lipopolysaccharide-binding protein (LBP) levels were not significantly reduced by artichoke extract monotherapy in either model, although combination therapy with probiotics and/or omega-3s led to significant reductions in LBP. These results support the use of both VPA and LPS as complementary models for studying ASD. The VPA model, characterized by direct and predictable neurotoxic effects, appears to be more suitable for evaluating preventive interventions. In contrast, the LPS model more accurately captures the complex immune-inflammatory mechanisms implicated in ASD, highlighting the need for broader and more individualized treatment strategies. The differing responses to artichoke-based interventions in these models underscore the importance of considering ASD etiology when designing dietary and microbiome-targeted therapies.

## 1 Introduction

Maternal health during pregnancy plays a leading role in shaping the health and disease risks of offspring. The earliest developmental stages are particularly susceptible to environmental insults, leading to significant and often irreversible consequences. Unfortunately, stress on the developing brain can have dramatic consequences for an individual’s neurodevelopment and future mental health. Maternal infection during pregnancy is associated with an increased risk of psychiatric and neurodevelopmental disorders (NDDs). Evidence suggests that maternal immune activation (MIA), independent of the infection itself, can be responsible for adverse outcomes in offspring [1], 2]. A cascade of pro-inflammatory cytokines and associated immunological alterations is actively transmitted across the placental barrier to the developing offspring. This prenatal immune challenge orchestrates lasting neuroimmune programming, leading to adverse neurodevelopmental and behavioral phenotypes, predominantly affecting the central nervous system (CNS) [3]. During embryonic development, maintenance of proper redox status is essential because redox processes play important roles in cell proliferation, apoptosis, and differentiation [4].

Induction of MIA with lipopolysaccharide (LPS) induces oxidative stress in the developing fetus, which may contribute to neurodevelopmental disorders. LPS is a component of Gram-negative bacterial cell walls that triggers a strong immune response, mimicking an infection during pregnancy. This immune response, or MIA, can lead to increased inflammation and oxidative stress in the fetus, potentially affecting brain development and autism spectrum disorder (ASD)-like behavior [5], 6].

Furthermore, valproic acid (VPA), commonly used to treat epilepsy and bipolar disorder, induces oxidative stress during pregnancy, potentially leading to birth defects. This oxidative stress, characterized by an imbalance between reactive oxygen species and antioxidant defenses, is believed to be a key mechanism in the development of neural tube defects and other congenital malformations associated with VPA exposure [7], 8].

Epidemiological data have identified prenatal exposure to specific environmental agents, including the anticonvulsant VPA and LPS, a trigger of MIA, as significant risk factors for ASD. Animal models also play a crucial role in ASD research by providing insights into the biological mechanisms underlying the disorder and potential treatments. Rodents are the most common animal models because of their genetic similarity to humans and their ability to exhibit behavioral traits relevant to ASD [9].

ASD is a complex neurodevelopmental syndrome that manifests as intrinsic impairments in social communication and interaction, coupled with restricted and repetitive patterns of behavior, interests, or activities. The designation “spectrum” acknowledges the considerable heterogeneity in the clinical presentation and severity of symptoms among affected individuals [10], [11], [12]. Data from the Centers for Disease Control and Prevention indicate a prevalence of approximately one in 31 children in the United States as of 2025 [13]. Globally, the World Health Organization reports an estimated prevalence of one in 100 children [14].

Elevated oxidative stress and impaired antioxidant defenses have been observed in individuals with ASD, potentially affecting neurodevelopment and contributing to behavioral symptoms. Studies have shown higher levels of oxidative stress markers, such as lipid peroxidation products, in individuals with ASD than in controls [15]. ASD Patients often exhibit reduced activity of antioxidant enzymes, such as glutathione reductase and glutathione peroxidase, and lower levels of reduced glutathione (GSH) [16]. Oxidative stress can disrupt various cellular processes, including mitochondrial function, neuroinflammation, and neurodevelopmental pathways, potentially contributing to the core features of ASD [17], 18].

The intestinal lining is composed of cells held together by tight junctions, which control the passage of substances from the gut into the bloodstream. Oxidative stress can disrupt these tight junctions, leading to increased permeability, commonly referred to as “leaky gut.” When the gut becomes leaky, substances that are normally contained within the gut, such as bacteria, toxins, and undigested food particles, can enter the bloodstream. This triggers an immune response, leading to inflammation. Inflammation caused by a leaky gut can affect the brain through the gut–brain axis, potentially contributing to neurodevelopmental issues and other symptoms associated with ASD [19], [20], [21].

In addition, the gut microbiome may also play a role, as disruptions in gut bacteria can lead to increased oxidative stress and inflammation in the gut, which can affect the brain [22], 23]. Notably, several studies suggest that addressing oxidative stress and improving gut health, potentially through dietary interventions and/or probiotic supplementation, may help reduce gut leakiness and improve outcomes in individuals with ASD [24], 25].

Probiotics (Pro), live microorganisms that confer health benefits to the host, have shown potential in mitigating oxidative stress associated with ASD. Research suggests that altered gut microbiota in individuals with ASD can contribute to increased oxidative stress in the gastrointestinal tract. Probiotics may help reduce oxidative stress and improve ASD-related symptoms by modulating the gut microbiota [26], 27]. In contrast, artichokes (AR) are considered a good source of prebiotics, primarily due to the presence of inulin, a type of fiber that acts as a substrate for beneficial gut bacteria. This prebiotic effect can improve digestion, nutrient absorption, and overall gut health [28]. Furthermore, artichokes are particularly rich in polyphenols, which are antioxidants. These polyphenols include cynarin and chlorogenic acid, which have been studied for their antioxidant and other beneficial properties [29]. The aim of the present study was to evaluate the potential ameliorative effects of a standardized polyphenol-rich extract derived from the leaves of *Cynara cardunculus* *L.* (artichoke), used as a prebiotic, either alone or in combination with a probiotic mixture and/or omega-3 (ω3) fatty acids, against LPS-induced MIA as well as VPA-induced oxidative stress and gut leakiness in a rat model of autism.

## 2 Materials and methods

### 2.1 Extract from *Cynara* *S* *colymus* *L.* (artichoke)

Soxhlet extraction of 10 kg of shade-dried powdered artichoke was performed using 96 % ethanol (4 × 1 L) for 3 h at 80 °C. The pooled extracts were clarified by filtering through Whatman No. 1 filter paper, and the solvent was subsequently removed under reduced pressure (approximately 40 °C) via rotary evaporation. The resulting dark green crude extract (101.7 g) was completely dried in a fume hood and stored at 4 °C for subsequent analysis. Artichoke extract was diluted in normal saline (0.9 % NaCl) to yield a dose of 400 mg/kg. The dose of artichoke was determined based on a literature review [30].

### 2.2 Mixture of probiotics

Infant probiotics (LoveBug Nutrition, NY), obtained from iHerb, consist of a mixture of healthy bacteria, including *Bifidobacterium infantis, Bifidobacterium lactis* and *Lactobacillus rhamnosus GG*, at a concentration of one billion CFU per 1.5 g [31].

### 2.3 Experimental design

Healthy female Wistar albino rats (8 ± 1 weeks old, 250 ± 15 g) were housed in polypropylene cages within an environmentally controlled clean-air room. The room was maintained at 25 °C (±1 °C), 50 % (±5 % RH), and a 12-h light/12-h dark cycle. For breeding, females were paired overnight with males (2:1 ratio), and the morning of confirmed sperm detection in the vaginal smear was designated as gestation day 1 (GD 1) (Table 1).

**Table 1:** Scheme of the animal experiments.

Group number	Intraperitoneal (IP) injection and timing	Oral administration of 400 mg/kg of artichoke extract
Pregnant females were randomly assigned to five experimental groups and were maintained on a standard diet, as follows:		
Group.1	Saline/12.5 GD	–
Group.2	600 mg/kg VPA/12.5 GD	–
Group.3	600 mg/kg VPA/12.5 GD	From 1st GD
Group.4	100 μg/kg LPS/9.5 GD	–
Group.5	100 μg/kg LPS/9.5 GD	From 1st GD

Group number	Group name	Oral administration
Females were housed individually and allowed to raise their own litters, and the experiments were performed on the male offspring		
Group.1.1	Control	Saline
Group.2.1	VPA-0	–
Group.2.2	VPA-AR	400 mg/kg artichoke extract
Group.2.3	VPA-AR.Pro	400 mg/kg of artichoke extract and a mixture of probiotics
Group.3.1	VPA.AR-AR	400 mg/kg artichoke extract
Group.4.1	LPS-0	–
Group.4.2	LPS-AR	400 mg/kg artichoke extract
Group.4.3	LPS-AR.Pro.ω3	400 mg/kg artichoke extract, and a mixture of probiotics with omega-3 fatty acids
Group.5.1	LPS.AR-AR	400 mg/kg artichoke extract

### 2.4 Ethical approval

All experimental procedures were conducted at the Centre for Experimental Surgery and Animals Lab, Prince Naif Health Research Centre, King Khalid University Hospital (KKUH), Riyadh, Saudi Arabia (SA).

**Ethical approval:** The research related to animal use has been complied with all the relevant national regulations and institutional policies for the care and use of animals, and has been approved by the Ethical Committee of Bioethics at King Saud University (KSU) (Ref No.: SE-24-33).

At the end of the experiment, plasma was collected from male pups using heparin as an anticoagulant for biochemical assays, and the rats were euthanized by inhalation of an overdose of sevoflurane. A 100 mg brain tissue sample was rinsed with pre-cooled 1X phosphate-buffered saline (PBS) obtained from Cepham Life Sciences (USA), homogenized using an IKA T18 Digital Ultra Turrax homogenizer (Germany) in 1 ml of 1X PBS, and stored overnight at −20 °C. After two freeze–thaw cycles were performed to disrupt the cell membranes, the homogenates were centrifuged for 5 min (10,000×*g,* 2–8 °C), and the resulting supernatants were collected. Store samples at −20 °C were centrifuged again after thawing before the assay. Likewise, plasma samples were immediately collected in heparin tubes and centrifuged for 15 min at 1,000×*g* and 2–8 °C. Plasma samples were stored in aliquots at −20 °C for later use.

– **Oxidative stress markers for brain analysis**
  Rat Lipid Peroxide (LPO), GSH and oxidized glutathione (GSSG) ELISA kits were purchased from ELK Biotechnology (ELK 8896, ELK 8577, and ELK 9195, respectively; Wuhan, China). All procedures were performed according to the manufacturer’s instructions. Brain homogenate samples were analyzed in duplicate.
– **Leaky gut markers for plasma analysis**
  Rat intestinal fatty acid binding protein (IFABP), lipopolysaccharide-binding protein (LBP), and occludin (OCLN) ELISA kits were purchased from ELK Biotechnology (ELK 2603, ELK 5655, and ELK 6134, respectively; Wuhan, China). The Zonulin ELISA kit was purchased from MyBioSource (MBS2606662, San Diego, USA). All procedures were performed according to the manufacturer’s instructions. Plasma samples were analyzed in duplicate.
– **Drugs and dosage**
  1. **Valproic acid**
    VPA salt was obtained from Sigma-Aldrich, United States (P4543 Sigma). The dose was calculated as 600 mg/kg body weight (120 mg/200 g body weight) and dissolved in normal saline (0.9 % NaCl) at a concentration of 250 mg/mL [32].
  2. **Lipopolysaccharide**
    LPS from *Escherichia coli* (serotype 055: B5, Solarbio, Beijing, China) was dissolved in saline solution at a concentration of 100 μg/kg (IP administration in a volume of 2 mL/kg on GD 9.5) [5].
  3. **Omega-3 polyunsaturated fatty acids (omega-3 PUFAs)**
    Omega-3 Premium Fish Oil (California Gold Nutrition, USA) was obtained from iHerb and administered orally at a dose of 200 mg/kg body weight/day [33].

### 2.5 Statistical analysis

Statistical analyses were conducted using the Statistical Package for the Social Sciences (SPSS; IBM, Chicago, IL, USA). Data are expressed as mean values (mean ± SD) for various biochemical markers across the nine experimental groups. Pairwise comparisons between all groups are shown for the following parameters: LPO, GSH, GSSG, IFABP, OCLN, zonulin and LBP. Significant one-way ANOVA was followed by multiple comparisons using the LSD test, whereas significant Kruskal–Wallis results were followed by Mann–Whitney tests, with significance indicated at *p < 0.05, **p < 0.01, and ***p < 0.001.

A heatmap using non-parametric Spearman’s correlation analysis (visually representing the strength and direction of relationships between pairs of variables using colors) was used to assess the relationships between biochemical parameters. For normally distributed data, positive and negative correlations were reported, with significance levels set at *p* ≤ 0.05 for moderate correlations and *p* ≤ 0.01 for strong correlations, with different colors indicating different correlation values.

Receiver Operating Characteristic (ROC) curve analysis was performed using SPSS to evaluate the diagnostic performance of the biochemical parameters. The true positive rate (sensitivity) was plotted against the false-positive rate (100 – specificity) across various cutoff points, and the area under the curve (AUC) was calculated to quantify diagnostic utility. Parameters with AUC values close to 1 were considered strong biomarker candidates in the VPA-0 and LPS-0 groups. The AUC values were interpreted as excellent (0.9–1.0), good (0.8–0.9), fair (0.7–0.8), poor (0.6–0.7), and very poor (0.5–0.6). In the treatment groups (VPA-AR, VPA-AR.Pro, VPA.AR-AR, LPS-AR, LPS-AR.Pro.ω3, and LPS.AR-AR), an AUC value of 0.7 or lower was considered indicative of treatment effectiveness because it suggested limited discrimination between the control and treatment groups for the biomarker.

## 3 Results

### 3.1 Biochemical alterations across experimental groups

**Figure 1:**
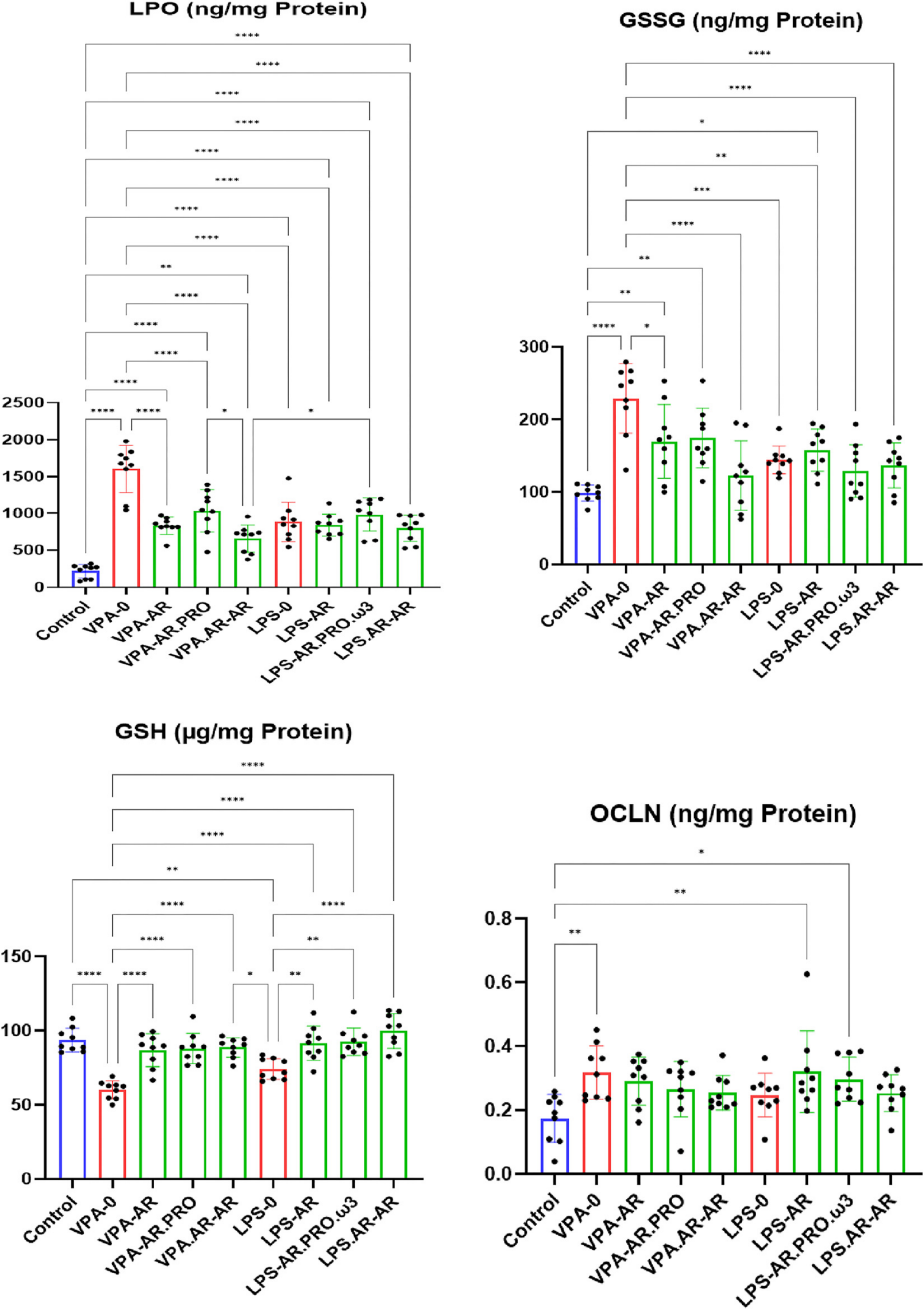

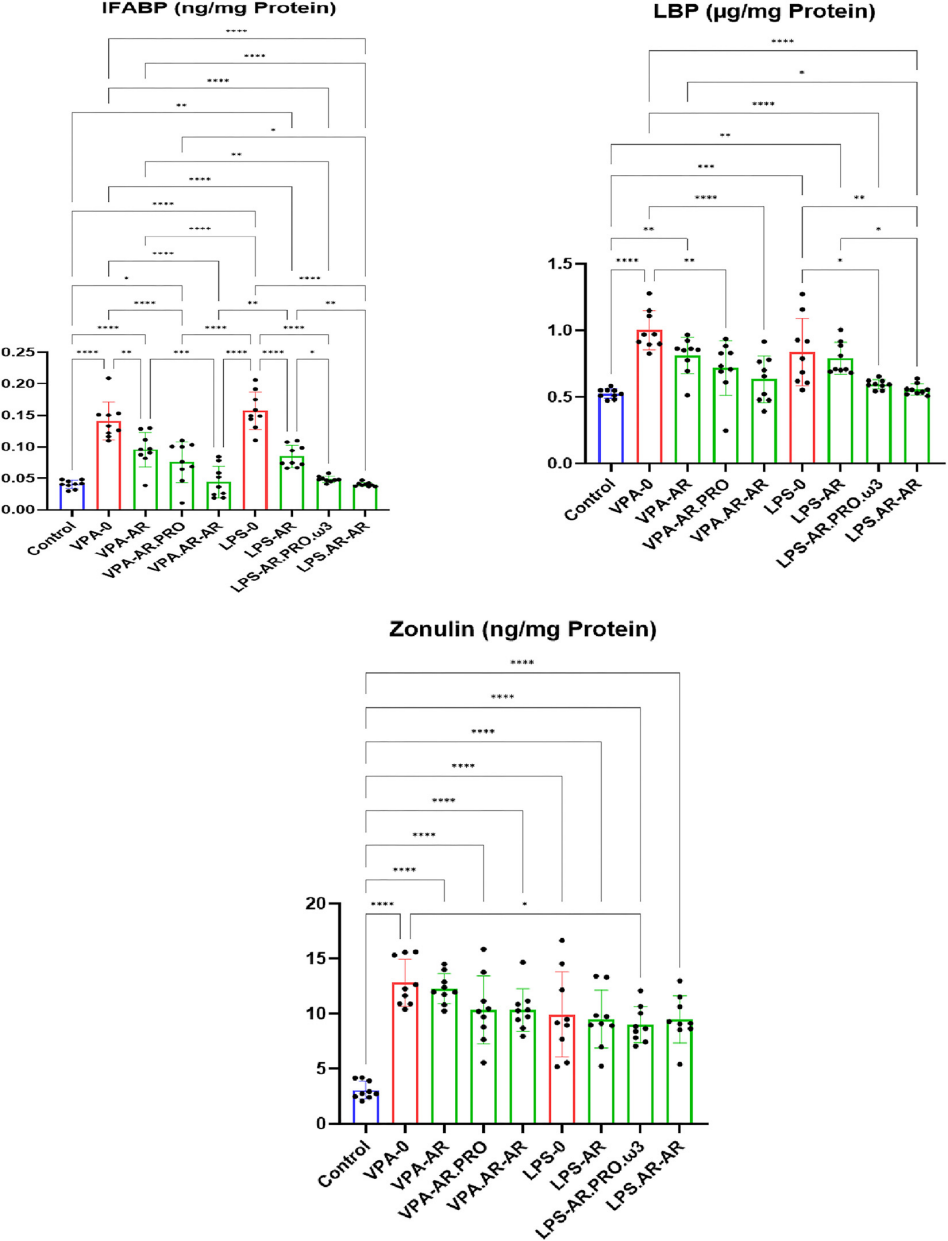
Various biochemical markers across nine experimental groups presented as mean ± SD values: control group (*n* = 9), VPA-0 group (*n* = 9), VPA-AR group (*n* = 9), and VPA-AR.Pro group (*n* = 9), VPA, AR-AR group (*n* = 9), LPS-0 group (*n* = 9), LPS-AR group (*n* = 9), LPS-AR.Pro.ω3 group (*n* = 9), and LPS.AR-AR group (*n* = 9). Pairwise comparisons between all groups are shown for the following parameters: LPO, GSSG, IFABP, OCLN, zonulin, and LBP. Significant one-way ANOVA result were followed by multiple comparisons using the LSD test, whereas significant Kruskal–Wallis results were followed by Mann–Whitney tests, with significance indicated at *p < 0.05, **p < 0.01, and ***p < 0.001. VPA: valproic acid, AR: artichoke, Pro: probiotics, ω3: omega-3, LPS: lipopolysaccharide, LPO: lipid peroxidation, GSH: glutathione, GSSG: glutathione disulfide, IFABP: intestinal fatty acid-binding protein, OCLN: occludin, and LBP: lipopolysaccharide-binding protein.

The results in Tables 2 and 3 and Figure 1 demonstrate significant biochemical alterations across the different treatment groups. The VPA-0 and LPS-0 groups showed increased levels of oxidative stress and intestinal leakiness biomarkers. In contrast, the levels of LPO, GSH, and IFABP were improved in the artichoke-treated group, as well as in the groups receiving combined treatment with probiotics and omega-3 fatty acids.

**Table 2:** Comparison between different groups for oxidative stress parameters.

Parameters	Groups	*N*	Min.	Max.	Mean ± SD	Percent change	*p* value
LPO (ng/mg protein)	Control	9	82.85	315.61	215.83 ± 90.00^a^	100.00	0.001
	VPA-0	9	1,044.83	1,979.63	1,604.49 ± 322.33^d^	743.41	
	VPA-AR	9	560.48	971.94	833.00 ± 117.09^bc^	385.95	
	VPA-AR.PRO	9	476.66	1,391.24	1,033.74 ± 287.87^c^	478.96	
	VPA.AR-AR	9	374.19	957.48	658.28 ± 187.12^b^	305.00	
	LPS-0	9	544.98	1,475.75	882.96 ± 269.25^bc^	409.10	
	LPS-AR	9	653.83	1,138.55	841.54 ± 150.02^bc^	389.91	
	LPS-AR.PRO.ω3	9	616.08	1,198.40	984.94 ± 226.06^c^	456.35	
	LPS.AR-AR	9	525.75	977.56	797.42 ± 179.91^bc^	369.47	
GSSG (ng/mg protein)	Control	9	75.40	111.13	98.40 ± 11.37^a^	100.00	0.001
	VPA-0	9	130.73	279.16	229.08 ± 48.06^c^	232.80	
	VPA-AR	9	99.90	252.84	169.73 ± 50.78^b^	172.49	
	VPA-AR.PRO	9	114.50	253.14	174.48 ± 40.88^bc^	177.32	
	VPA.AR-AR	9	62.27	195.47	122.86 ± 47.86^ab^	124.86	
	LPS-0	9	119.31	187.74	144.43 ± 19.26^ab^	146.77	
	LPS-AR	9	111.03	194.53	158.01 ± 29.09^b^	160.58	
	LPS-AR.PRO.ω3	9	90.30	193.64	128.90 ± 36.37^ab^	130.99	
	LPS.AR-AR	9	85.38	174.97	136.52 ± 30.98^ab^	138.74	
GSH (µg/mg protein)	Control	9	85.30	108.25	93.42 ± 8.04^c^	100.00	0.001
	VPA-0	9	50.08	69.36	60.19 ± 6.08^a^	64.43	
	VPA-AR	9	66.65	99.35	86.83 ± 11.00^bc^	92.94	
	VPA-AR.PRO	9	76.56	109.47	87.94 ± 10.28^bc^	94.13	
	VPA.AR-AR	9	76.18	96.35	88.66 ± 6.55^c^	94.91	
	LPS-0	9	65.99	83.62	74.11 ± 6.97^ab^	79.33	
	LPS-AR	9	72.42	111.94	91.40 ± 11.57^c^	97.84	
	LPS-AR.PRO.ω3	9	82.61	112.58	92.43 ± 9.20^c^	98.93	
	LPS.AR-AR	9	82.54	113.60	99.65 ± 11.80^c^	106.67	

Groups with different letters are significantly different from each other at the 0.05 significance level (*p* < 0.05). Groups with the same letter are not significantly different from each other.

**Table 3:** Comparison between different groups for intestinal leakiness parameters.

Parameters	Groups	*N*	Min.	Max.	Mean ± SD	Percent change	*p* value
OCLN (ng/mg protein)	Control	9	0.10	0.26	0.19 ± 0.06^a^	100.00	0.004
	VPA-0	9	0.23	0.45	0.32 ± 0.08^bd^	167.76	
	VPA-AR	9	0.16	0.38	0.29 ± 0.08^bcd^	153.76	
	VPA-AR.PRO	9	0.20	0.35	0.29 ± 0.05^bcd^	151.95	
	VPA.AR-AR	9	0.21	0.37	0.25 ± 0.05^abc^	134.37	
	LPS-0	9	0.11	0.36	0.25 ± 0.07^ab^	130.72	
	LPS-AR	9	0.20	0.39	0.29 ± 0.06^bc^	151.31	
	LPS-AR.PRO.ω3	9	0.22	0.38	0.30 ± 0.07^bcd^	157.04	
	LPS.AR-AR	9	0.14	0.33	0.25 ± 0.06^ab^	133.88	
Zonulin (ng/mg protein)	Control	9	2.06	4.19	3.06 ± 0.81^a^	100.00	0.001
	VPA-0	9	10.40	15.62	12.80 ± 2.14^c^	417.59	
	VPA-AR	9	10.25	14.48	12.26 ± 1.37^bc^	400.22	
	VPA-AR.PRO	9	5.56	15.85	10.34 ± 3.07^bc^	337.56	
	VPA.AR-AR	9	7.96	14.66	10.34 ± 1.91^bc^	337.49	
	LPS-0	9	5.19	16.64	9.93 ± 3.86^bc^	324.08	
	LPS-AR	9	5.24	13.39	9.50 ± 2.62^bc^	310.09	
	LPS-AR.PRO.ω3	9	7.06	12.07	9.00 ± 1.63^b^	293.58	
	LPS.AR-AR	9	5.41	12.98	9.46 ± 2.12^bc^	308.76	
IFABP (ng/mg protein)	Control	9	0.03	0.05	0.041 ± 0.006^a^	100.00	0.001
	VPA-0	9	0.11	0.21	0.141 ± 0.030^d^	347.38	
	VPA-AR	9	0.04	0.13	0.096 ± 0.027^c^	235.47	
	VPA-AR.PRO	9	0.01	0.11	0.076 ± 0.032^bc^	186.24	
	VPA.AR-AR	9	0.02	0.09	0.044 ± 0.025^ab^	109.04	
	LPS-0	9	0.11	0.21	0.157 ± 0.030^d^	386.59	
	LPS-AR	9	0.07	0.11	0.085 ± 0.018^c^	208.97	
	LPS-AR.PRO.ω3	9	0.04	0.06	0.049 ± 0.005^ab^	119.55	
	LPS.AR-AR	9	0.04	0.05	0.040 ± 0.003^a^	99.16	
LBP (µg/mg protein)	Control	9	0.47	0.58	0.53 ± 0.04^a^	100.00	0.001
	VPA-0	9	0.83	1.28	1.00 ± 0.15^d^	190.31	
	VPA-AR	9	0.51	0.97	0.81 ± 0.14^bcd^	154.25	
	VPA-AR.PRO	9	0.25	0.94	0.72 ± 0.20^abc^	136.48	
	VPA.AR-AR	9	0.39	0.92	0.63 ± 0.17^abc^	120.14	
	LPS-0	9	0.55	1.27	0.84 ± 0.25^cd^	158.94	
	LPS-AR	9	0.68	1.00	0.79 ± 0.12^bcd^	150.03	
	LPS-AR.PRO.ω3	9	0.54	0.65	0.59 ± 0.03^ab^	112.88	
	LPS.AR-AR	9	0.51	0.64	0.56 ± 0.04^a^	105.87	

Groups with different letters are significantly different from each other at the 0.05 significance level (*p* < 0.05). Groups with the same letter are not significantly different from each other.

### 3.2 Correlations between biochemical parameters

**Figure 2:**
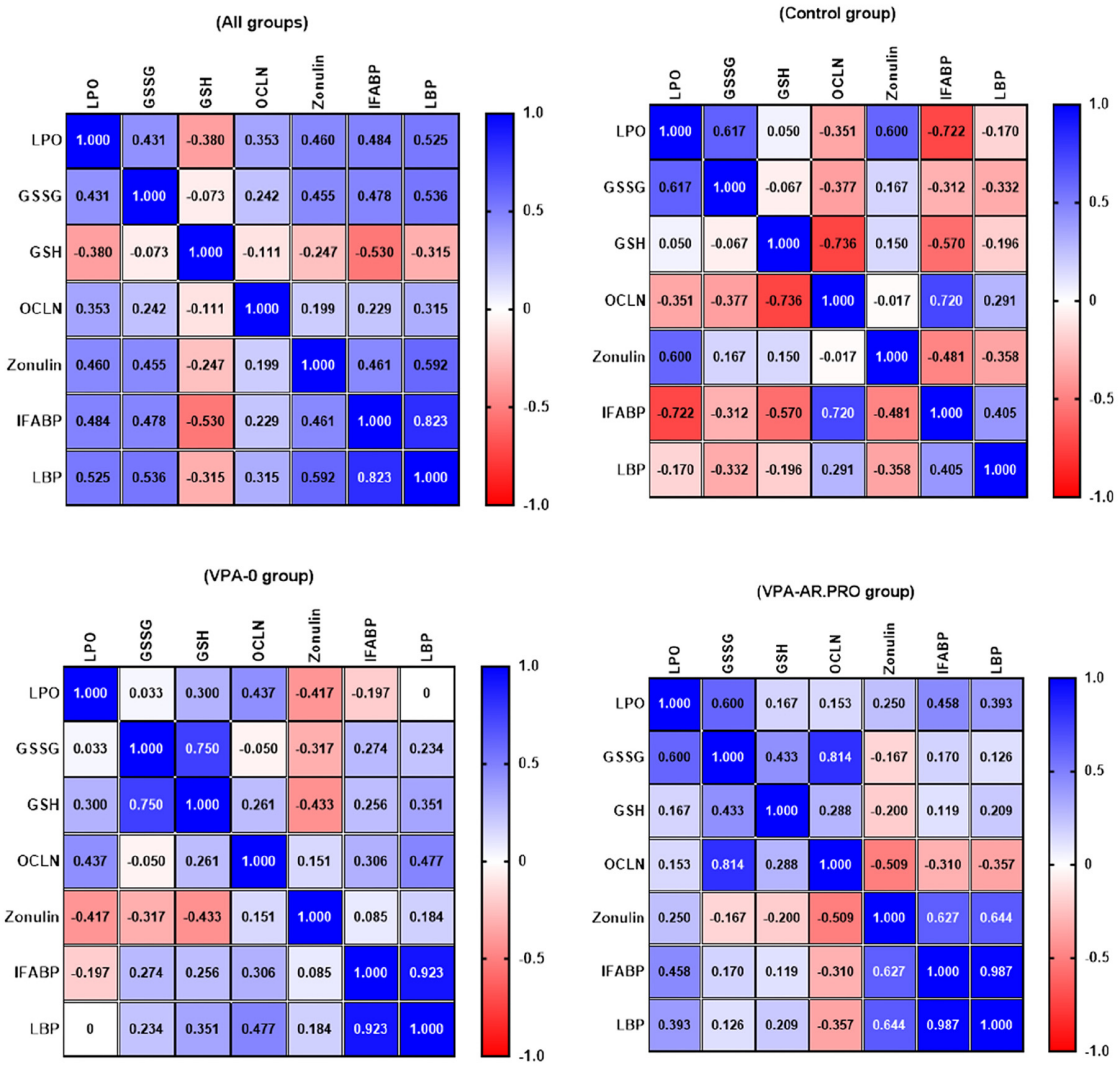

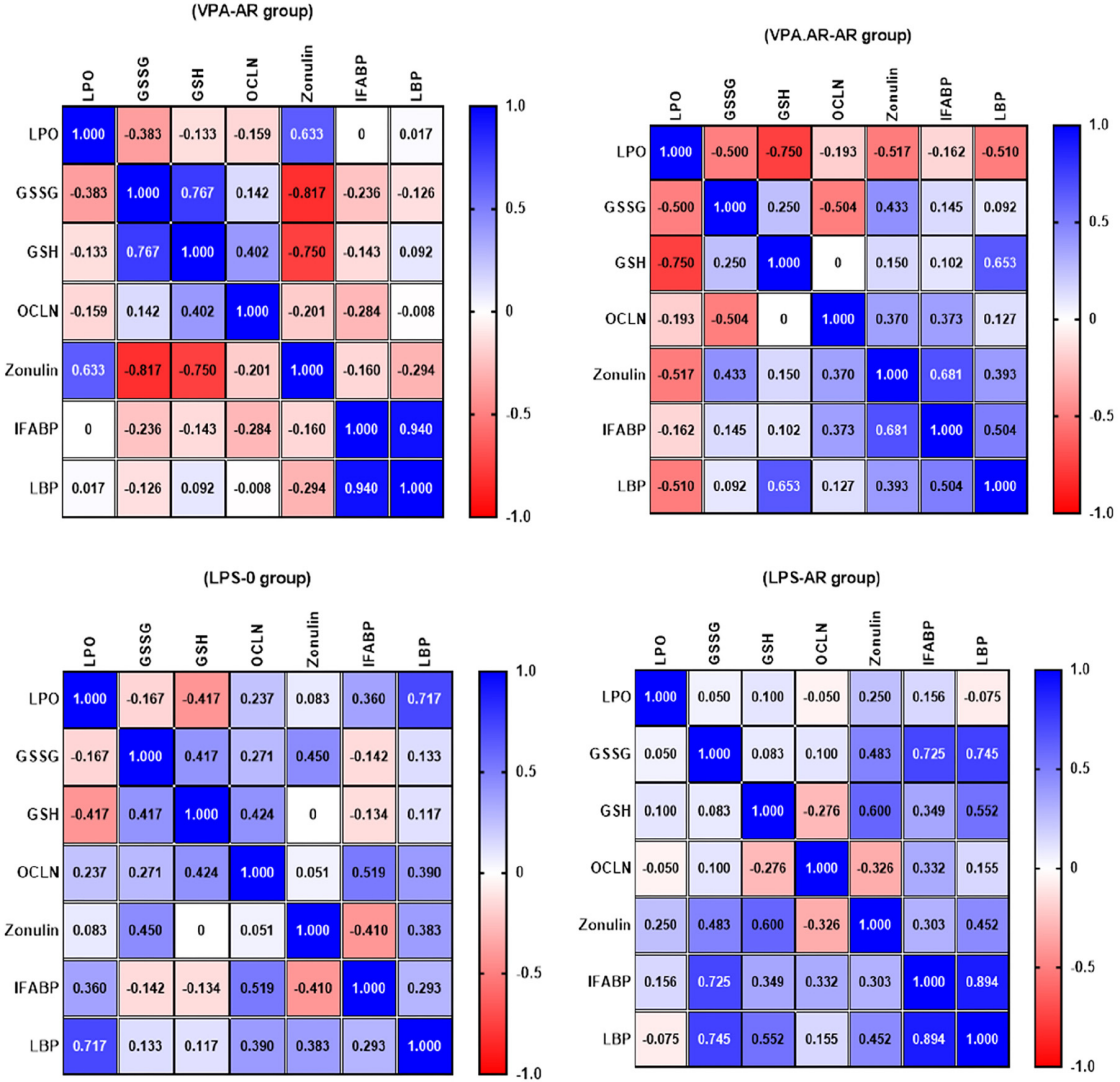

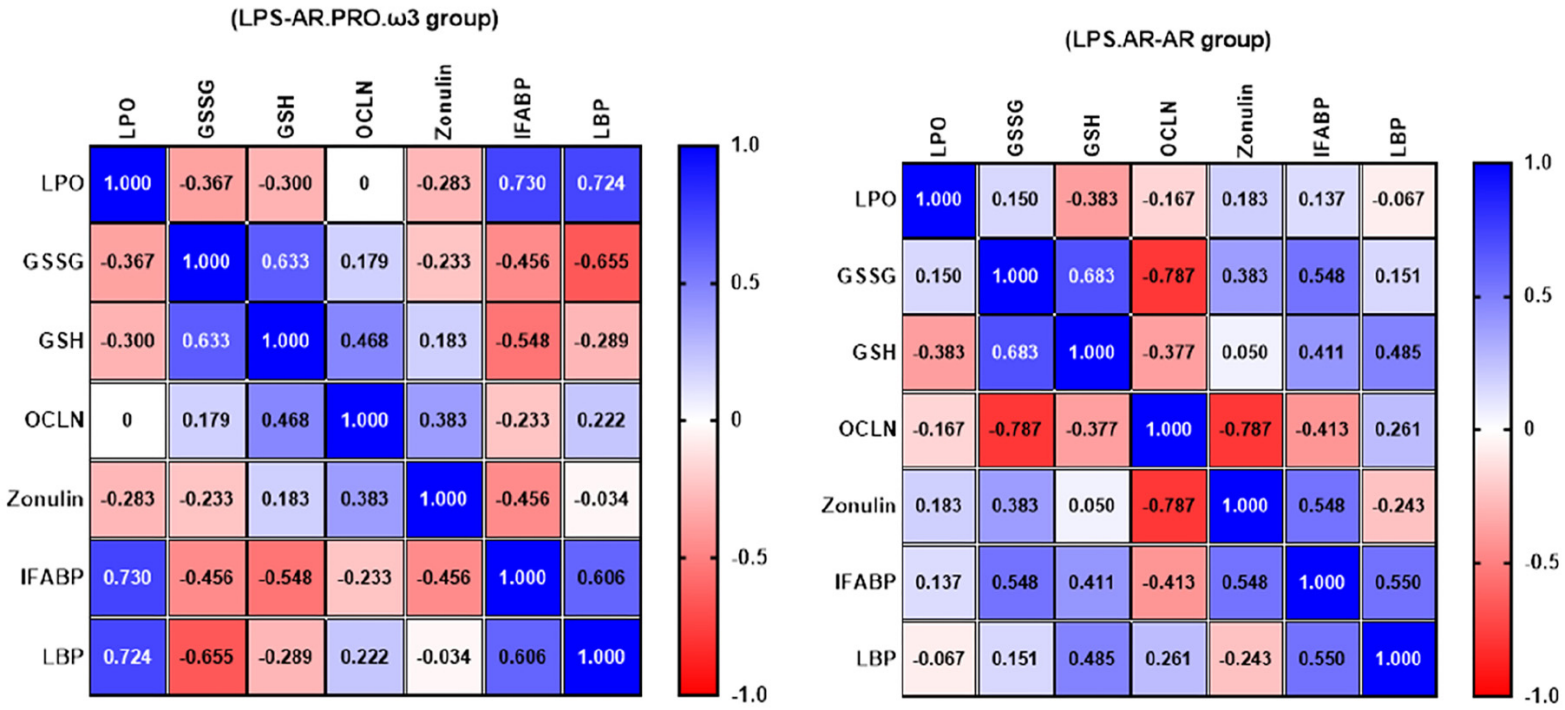
Heatmaps of non-parametric Spearman’s correlation between the measured parameters.

The results in Figure 2 revealed significant correlations between various oxidative stress and intestinal leakiness biomarkers. There was a positive correlation between LPO and GSSG and intestinal leakiness biomarkers, whereas LPO and GSSG showed a negative correlation with the antioxidant GSH. In contrast, GSH showed a negative correlation with intestinal leakiness markers. Positive correlations were also observed between the intestinal leakiness biomarkers.

### 3.3 ROC analysis of biochemical parameters in experimental groups

**Figure 3:**
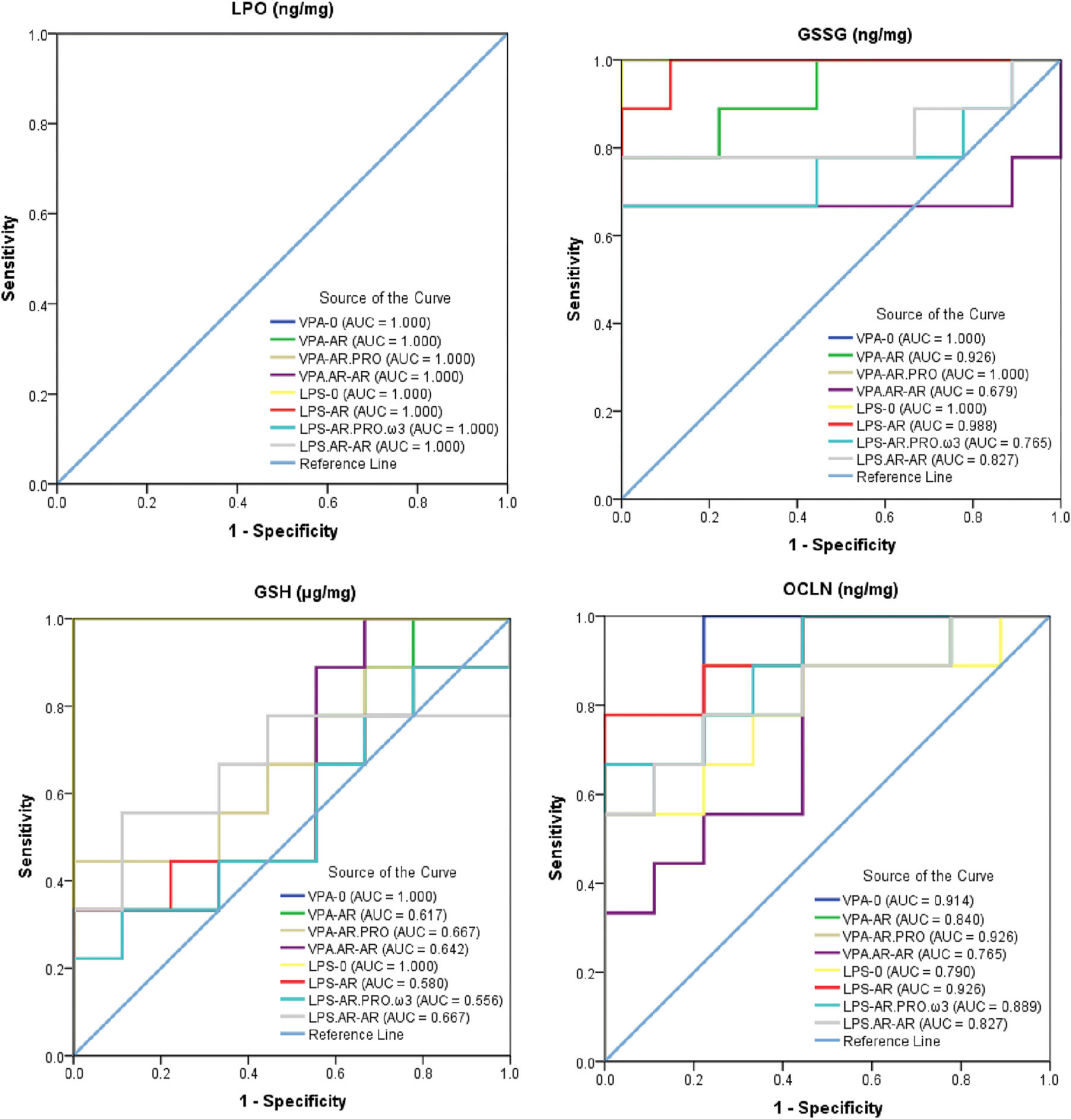

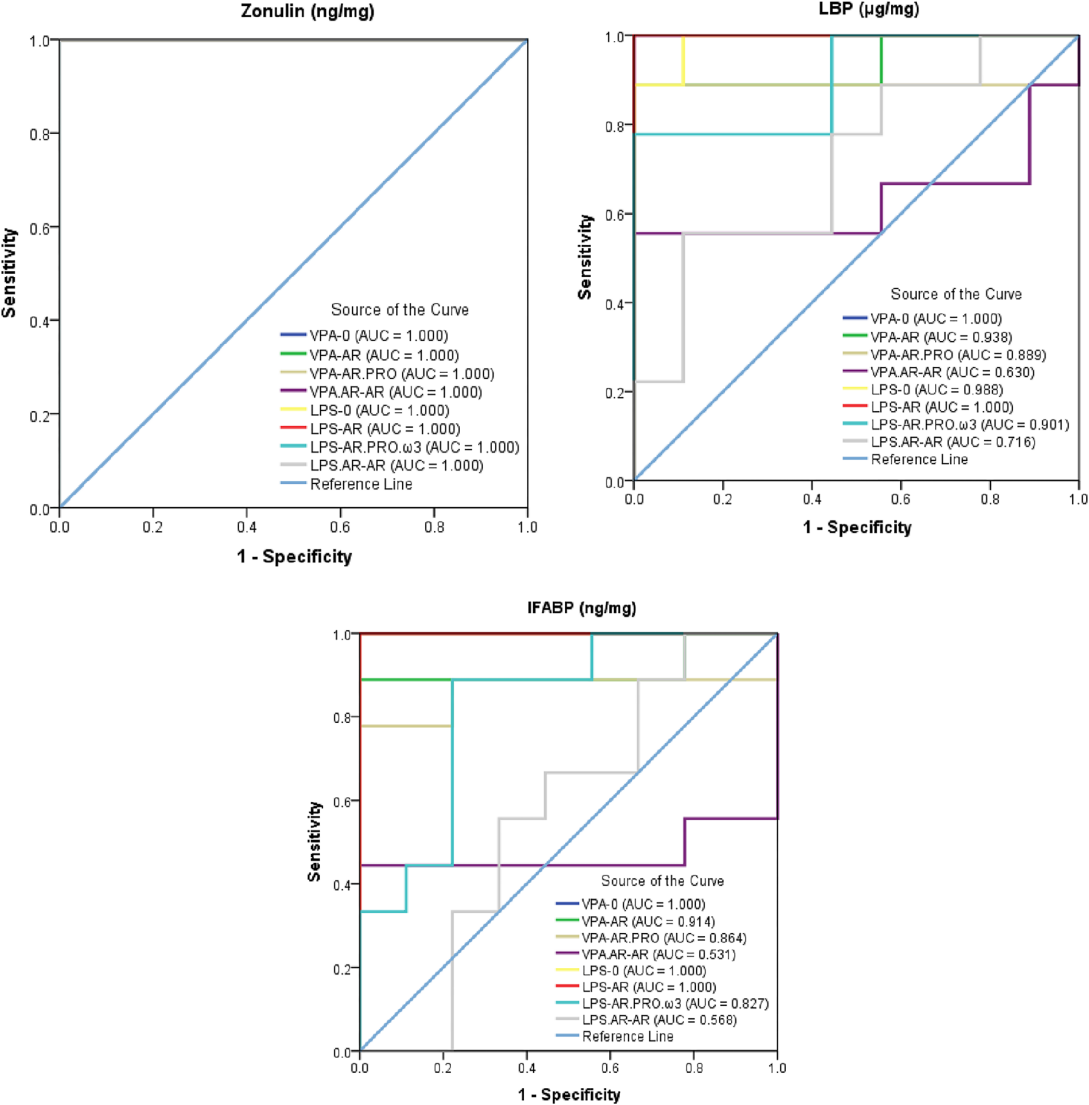
ROC curves for the measured variables in all the studied groups, using the control group as a reference group.

The ROC analysis in Figure 3 showed higher AUC scores (0.7–1.0) for oxidative stress and intestinal leakiness biomarkers in the VPA-0 and LPS-0 groups using the control group as a reference. In the treatment groups (VPA-AR, VPA-AR.Pro, VPA.AR-AR, LPS-AR, LPS-AR.Pro.ω3, and LPS.AR-AR), lower AUC scores were observed using the control group as a reference.

## 4 Discussion

To overcome the limitations of clinical studies and elucidate the molecular underpinnings of ASD, there is a pressing need to develop appropriate rodent models that mimic human ASD for detailed mechanistic investigations. The current findings demonstrated that prenatal exposure to both VPA and LPS was significantly associated with MIA, oxidative stress, and impaired intestinal barrier function, commonly referred to as “leaky gut”, thereby increasing the risk of ASD and neurodevelopmental abnormalities in offspring [18], [34], [35], [36]. As shown in Table 2 and Figure 1, LPO and GSSG levels increased in the prenatal VPA- and LPS-exposure groups (VPA-0 and LPS-0), with a significant decrease in the antioxidant glutathione in the brain homogenate. Furthermore, Table 3 and Figure 1 show elevated plasma levels of zonulin, a protein that regulates tight junction permeability; OCLN, a transmembrane protein that serves as a structural component of tight junctions; and IFABP, which is exclusively expressed in the epithelial cells of the small intestine, indicating a disrupted intestinal barrier. These alterations were positively correlated with ASD-like symptoms. Additionally, increased LBP levels, a marker of bacterial translocation from the gut, suggest enhanced intestinal permeability [37]. This condition is linked to food sensitivities and systemic inflammation and has been observed in rodent models of prenatal VPA and LPS exposure [36], 38].

The association between oxidative stress and leaky gut in MIA models induced by LPS and in models involving prenatal exposure to VPA has been supported by multiple studies. These studies reported an imbalance in redox homeostasis, a compromised intestinal barrier, and their involvement in the pathogenesis of ASD [39], [40], [41], [42]. *In utero* exposure to VPA is a well-established environmental risk factor for ASD, and oxidative stress plays a key role in VPA-induced neurotoxicity. This includes synaptic dysfunction and impaired neurogenesis [41], 43]. Researchers have reported that VPA-induced ASD-like behaviors are accompanied by significant thinning of the intestinal mucosa (epithelial lining) and muscularis layers [42].

Maternal immune responses can further exacerbate oxidative stress through inflammation-driven production of reactive oxygen species and mitochondrial dysfunction, affecting the mother, placenta, and fetus [18], 44]. Increased intestinal permeability and immune dysregulation observed in MIA offspring mirror findings in subsets of individuals with ASD, supporting a potential link between prenatal inflammation and neurodevelopmental outcomes [45], 46]. Furthermore, a leaky gut allows pro-inflammatory substances to enter the bloodstream and reach the brain, contributing to systemic inflammation, neuroinflammation, and increased oxidative stress [47], 48].

Identifying and measuring specific biomarkers of oxidative stress and impaired intestinal barrier function could serve as valuable tools for the early diagnosis and monitoring of therapeutic intervention efficacy in ASD. In the present study, we investigated the effects of artichoke-derived prebiotics, both alone and in combination with probiotics and omega-3 fatty acids, on alleviating symptoms in a rat model of induced autism.

Artichoke-derived inulin is characterized by a high degree of polymerization, which enhances its prebiotic efficacy and persistence in the colon. Upon microbial fermentation, inulin yields short-chain fatty acids (SCFAs), including acetate, propionate, and butyrate, which lower colonic pH and foster a gut environment conducive to the growth of beneficial bacteria, such as *Bifidobacterium* and *Lactobacillus*. These microbial shifts can suppress the growth of pathogenic species and exert immunomodulatory effects. Clinical studies have demonstrated that the prebiotic effects of artichokes are primarily mediated by increased populations of *Bifidobacterium* in the gut. Additionally, artichokes are rich in phenolic acids derived from caffeoylquinic acid, flavonoids such as luteolin and apigenin, and minerals and vitamins, which synergistically contribute to their health benefits. These polyphenols support antioxidant activity and contribute to digestive and immune health [28], 29], 49], 50].

As shown in Table 2 and Figure 1, there was a significant decrease in LPO and GSSG levels in the brains of the VPA-artichoke postnatal (VPA-AR) and VPA-artichoke with probiotics postnatal (VPA-AR.Pro), and VPA-artichoke pre/postnatal (VPA.AR-AR) group. These reductions were accompanied by a significant increase in GSH levels compared with those in the VPA-exposed control group. These findings suggest a beneficial association between prebiotic intervention – either alone or in combination with probiotics – and reduced oxidative stress in VPA-exposed offspring. Prebiotics have demonstrated potential as a therapeutic strategy to mitigate VPA-induced neurotoxicity by improving the redox balance and decreasing oxidative stress. This neuroprotective effect is likely mediated indirectly through improved gut health, which reduces systemic inflammation and, in turn, neuroinflammation in the central nervous system [51], [52], [53].

Previous studies have also shown that synbiotic interventions (i.e., combined prebiotics and probiotics) can alleviate a range of ASD-like symptoms in rodent models exposed to VPA. These therapeutic benefits include improvements in behavioral outcomes, modulation of inflammatory cytokines such as IL-6 and IL-10, regulation of key neurotransmitter systems – including serotonin and GABA – and restoration of gut microbiota homeostasis [30], 54], 55]. Artichoke extracts are rich in bioactive phytochemicals, particularly phenolic and flavonoid compounds, with hydroxycinnamic acids derived from caffeic acid being the predominant phenolic constituents. These compounds are primarily present in conjugation with quinic acid, forming caffeoylquinic acid derivatives, among which cynarin (1,3-O-dicaffeoylquinic acid), chlorogenic acid (3-O-caffeoylquinic acid), neochlorogenic acid (5-O-caffeoylquinic acid), and 1,5-O-dicaffeoylquinic acid are the most notable [56]. Flavonoids are the second most abundant class, with flavones, such as apigenin, luteolin, and their glycoside and rutinoside derivatives, being particularly prominent. They possess substantial antioxidant capacity, facilitating the direct scavenging of reactive oxygen and nitrogen species (ROS/RNS) and mitigating subsequent macromolecular oxidative damage. *In vitro* evaluation demonstrated that administration of *C.* *S* *colymus* extract significantly attenuated oxidative stress markers, specifically in hydrogen peroxide (H_2_O_2_)-induced human hepatoma (HepG2) cells. Notably, treatment with the extract suppressed intracellular ROS accumulation and upregulated key endogenous antioxidant enzymes, including superoxide dismutase and catalase, thereby enhancing cellular resilience to oxidative insults [18], 29], 53], 57].

Furthermore, plasma intestinal IFABP concentrations were significantly reduced in the VPA-AR, VPA-AR.Pro, and VPA.AR-AR groups. This reduction suggests that prebiotic and synbiotic interventions may promote intestinal health, thereby limiting enterocyte damage and reducing IFABP release into circulation, which may represent a potential protective mechanism against conditions associated with elevated IFABP levels [58].

In contrast, plasma levels of the intestinal permeability biomarkers zonulin and occludin were reduced nonsignificantly across the treatment groups. This indicates that prebiotic intervention – whether administered alone or in combination with probiotics – was insufficient to restore gut barrier function impaired by VPA exposure. This finding is supported by Ng [59], who concluded that prebiotics and probiotics have shown limited efficacy in managing gastrointestinal symptoms in children with ASD, despite encouraging preclinical data.

Prince et al. (2024) reported the normalization of immune and intestinal permeability deficits in a VPA-induced ASD mouse model following a 3 % galacto-oligosaccharides and fructo-oligosaccharides prebiotic diet. However, the present study did not observe a similar corrective effect with artichoke-derived inulin. The lack of efficacy may be attributed to differences in experimental protocols, including the choice of prebiotic agent, dosage (400 mg/kg), and duration and timing of intervention. Notably, the current study employed a shorter intervention period (23 days from PND 7) than the longer early-life supplementation period (49 days from birth) used by Prince et al. [60].

Additionally, the findings of the present study contrast with those reported by Li X et al. (2024), who demonstrated that a 1 % inulin fiber diet effectively normalized immune dysregulation and intestinal barrier deficits in a model of EHDPHP-induced intestinal toxicity. In our study, artichoke-derived prebiotic monotherapy (400 mg/kg) did not ameliorate VPA-induced intestinal permeability impairment. This discrepancy may be attributed to key methodological differences, including variations in prebiotic type, dosage, and treatment duration. Notably, Li X et al. implemented a longer intervention period (8 weeks) than the 4-week duration in this study, although both studies began treatment on postnatal day 7 [61].

In contrast, levels of LBP, a critical component of the innate immune response to Gram-negative bacterial infections, were significantly reduced in the VPA-AR.Pro and VPA.AR-AR groups and showed a nonsignificant reduction in the VPA-AR group, as shown in Table 3 and Figure 1. This suggests that prebiotic and synbiotic interventions may modulate LBP levels, potentially by enhancing gut barrier integrity and reducing endotoxin (LPS) translocation from the gut into the bloodstream. Although direct modulation of LBP is not the primary target of most gut-focused therapies, emerging evidence indicates that improving gut permeability through prebiotic and probiotic interventions can indirectly reduce circulating LPS levels, thereby attenuating LBP-mediated immune responses. Specific probiotic strains, such as *Bifidobacterium* and *Akkermansia*, in conjunction with prebiotics, have shown potential for lowering LPS levels and improving gut–immune axis function [62], 63].

In the MIA model induced by LPS, no significant improvements were observed in the oxidative stress biomarkers LPO and GSSG following treatment with artichoke as a prebiotic – administered either alone pre/postnatally (LPS-AR) or in combination with probiotics and omega-3 fatty acids (LPS-AR.Pro.ω3 and LPS.AR-AR). However, a significant increase in GSH levels was observed in these groups compared with the LPS-only exposure group, as shown in Table 2 and Figure 1. Additionally, plasma concentrations of intestinal permeability markers zonulin and OCLN exhibited only nonsignificant changes, suggesting that the artichoke-based intervention, whether as a monotherapy or in combination with probiotics and omega-3, did not effectively reverse LPS-induced oxidative stress or intestinal barrier dysfunction.

These findings contrast with those reported by Shreya and Mohanty (2024), who demonstrated that fructooligosaccharide (FOS) supplementation significantly modulated LPS-induced oxidative stress in a dose-dependent manner (2 and 4 g/kg). Their study reported decreased oxidative stress markers and enhanced antioxidant defense, including increased GSH levels. The discrepancy between the current results and those of Shreya and Mohanty may be attributed to methodological differences, particularly the lower prebiotic dosage used in this study (400 mg/kg) and the shorter treatment duration (23 days from postnatal day 7) [64].

Despite these limitations, a significant reduction in plasma intestinal IFABP levels was observed in all treatment groups (LPS-AR, LPS-AR.Pro.ω3, and LPS.AR-AR), indicating an improvement in intestinal epithelial health. This reduction suggests that the intervention may have helped preserve gut integrity and limited epithelial damage, as evidenced by the lower release of IFABP into the circulation – a recognized biomarker of enterocyte injury [58], 65].

In contrast, LBP levels were significantly reduced in the LPS-AR.Pro.ω3 and LPS.AR-AR groups and nonsignificantly in the LPS-AR group, as shown in Table 3 and Figure 1. Omega-3 fatty acids, prebiotics, and probiotics show promise in modulating LBP responses. This is because they can enhance gut barrier integrity, thereby limiting the translocation of LPS into the bloodstream. Although these substances may not directly act on LBP, their ability to indirectly lower circulating LPS levels can influence LBP activation [62], 63], 66], 67].

In summary, VPA and LPS are widely used to model ASD in preclinical studies, albeit via distinct mechanisms. VPA induces ASD-like phenotypes primarily through direct neurotoxic and teratogenic effects, whereas LPS triggers MIA, leading to neurodevelopmental disruption via immune-mediated pathways. These different mechanisms present unique challenges in designing and evaluating therapeutic interventions.

In the VPA model, artichoke-derived prebiotic treatment – administered either prenatally, postnatally, or in combination with probiotics – effectively improved oxidative stress markers, as indicated by decreased levels of LPO and GSSG, alongside a significant increase in GSH. In contrast, similar interventions in the LPS-induced MIA model failed to significantly ameliorate oxidative stress, despite a modest increase in GSH levels. This suggests that oxidative stress resulting from VPA exposure is more responsive to dietary antioxidant interventions than immune-driven oxidative stress observed in the LPS model. A decrease in oxidative stress has been associated with a considerable increase in social interactions (Alaskar et al., under publication) [68].

Regarding gut permeability, plasma levels of the biomarkers zonulin and OCLN did not show significant improvement in either model following treatment with artichokes alone or in combination with probiotics and omega-3 fatty acids. However, IFABP levels, a sensitive marker of enterocyte injury, were significantly reduced in all treatment groups across both models, indicating epithelial protection and improved intestinal health.

LBP, a marker of systemic immune activation due to bacterial endotoxin translocation, was not significantly reduced by artichoke extract monotherapy in either model. However, combination therapy with probiotics and/or omega-3 fatty acids led to significant reductions in LBP, highlighting the importance of multimodal interventions in addressing immune-related components of ASD.

Figure 2 revealed a positive association between oxidative stress and compromised intestinal barrier indicators, whereas both were inversely correlated with GSH. The most notable finding was the substantial negative correlation between zonulin, a marker of increased intestinal permeability, and GSH and GSSG (the oxidized form of GSH) in the VPR-AR group (Figure 2). This supports AR’s antioxidant action and the role of GSH in reducing the elevated amount of zonulin, which is a marker of increased intestinal permeability or leaky gut.

In the LPS groups, which are considered a model of MIA-induced ASD-features, fewer significant negative associations were observed between zonulin and GSH (Figure 2). This suggests that in MIA, oxidative stress and gut barrier dysfunction are more severe and potentially less responsive to AR treatment than those caused by VPA exposure. This can be supported by the work of Liu K. et al. (2023), who reported that maternal infection contributed to an increased severity of ASD symptoms [69].

The AUC is a quantitative metric used in rodent models to indicate the overall change in physiological or biological variables under different experimental conditions, such as disease induction, drug exposure, and treatment strategies. This single value summarizes the entire curve, allowing for easy comparisons of outcomes across groups and treatments [70]. All biomarkers in the VPA and LPS exposure groups (VPA-0, LPS-0) exhibited high AUC values of 1.0 or approximately 1.0, confirming the severe biochemical alterations and disruption caused by VPA and LPS (i.e., high discriminating power). In contrast, the artichoke-only group showed high to moderate ameliorative effects across most parameters, with AUC values generally around 0.7, indicating less discriminating power compared to the control, or, in other words, improvements in impaired antioxidant capacity and increased intestinal permeability caused by VPA and LPS exposure (Figure 3).

## 5 Conclusions

Overall, these findings support the validity of both VPA and LPS as complementary models for studying ASD. However, the VPA model may be more amenable to preventive interventions because of its direct and more predictable neurotoxic effects. In contrast, the LPS model reflects the complexity of immune-inflammatory contributions to ASD, which may require more tailored and comprehensive treatment strategies. The differing responses to artichoke-based interventions underscore the need to consider the underlying etiology of ASD when developing dietary or microbiome-targeted therapies, with artichoke representing a promising source of bioactive compounds for neuroprotective and anti-inflammatory applications. However, it is important to note that a limitation of the present study was the exclusive use of male animals, based on the higher prevalence of ASD in male children. This approach may restrict our understanding of sex-specific differences in rodent models. Including both sexes in future studies would offer a more comprehensive perspective and help elucidate the potential pathways involved in the etiology and pathogenesis of the disorder. In addition, integrating metagenomic and metabolomic analyses would provide highly resolved mechanistic insights into both taxonomic composition and active metabolic pathways underlying the observed phenotypic and metabolic alterations. Furthermore, the relatively short intervention duration precluded the evaluation of the long-term efficacy of *C. scolymus* (artichoke) on metabolic homeostasis and gut microbiota composition. Consequently, longitudinal clinical trials featuring extended intervention periods are warranted to ascertain the sustainability of these physiological outcomes and to characterize long-term safety and efficacy profiles.

## Supplementary Material

Supplementary Material
