# Supplementary material for: Effects of artichoke extracts and probiotic on maternal immunological activation, valproic acid-induced oxidative stress, and gut leakiness in a rat model of autism
Source: Open Life Sci. 2026 Aug 18;21(1):20251355. doi: 10.1515/biol-2025-1355 (PMC13478924; doi:10.1515/biol-2025-1355)
Supplement: Supplementary file 1 [file j_biol-2025-1355_suppl_001.docx]

**Effects of Artichoke Extracts and Probiotic on Maternal Immunological Activation, Valproic Acid-Induced Oxidative Stress, and Gut Leakiness in a Rat Model of Autism**

**Supplementary Material**

1 **Supplementary Figures and Tables**

**Table 3.1**

| **Table 3. 1. Comparison between different groups for the oxidative stress parameters, One-way ANOVA test between different groups with multiple comparisons (Tukey test) within the entire groups.** | | | | | | | |
| --- | --- | --- | --- | --- | --- | --- | --- |
| **Parameters** | **Groups** | **N** | **Min.** | **Max.** | **Mean ± S.D.** | **Percent change** | **P value** |
| LPO (ng/mg Protein) | Control | 9 | 82.85 | 315.61 | 215.83 ± 90.00 **^a^** | 100.00 | 0.001 |
|  | VPA-0 | 9 | 1044.83 | 1979.63 | 1,604.49 ± 322.33 **^d^** | 743.41 |  |
|  | VPA-AR | 9 | 560.48 | 971.94 | 833.00 ± 117.09 **^bc^** | 385.95 |  |
|  | VPA-AR.PRO | 9 | 476.66 | 1391.24 | 1033.74 ± 287.87 **^c^** | 478.96 |  |
|  | VPA.AR-AR | 9 | 374.19 | 957.48 | 658.28 ± 187.12 **^b^** | 305.00 |  |
|  | LPS-0 | 9 | 544.98 | 1475.75 | 882.96 ± 269.25 **^bc^** | 409.10 |  |
|  | LPS-AR | 9 | 653.83 | 1138.55 | 841.54 ± 150.02 **^bc^** | 389.91 |  |
|  | LPS-AR.PRO.ω3 | 9 | 616.08 | 1198.40 | 984.94 ± 226.06 **^c^** | 456.35 |  |
|  | LPS.AR-AR | 9 | 525.75 | 977.56 | 797.42 ± 179.91 **^bc^** | 369.47 |  |
| GSSG (ng/mg Protein) | Control | 9 | 75.40 | 111.13 | 98.40 ± 11.37 **^a^** | 100.00 | 0.001 |
|  | VPA-0 | 9 | 130.73 | 279.16 | 229.08 ± 48.06 **^c^** | 232.80 |  |
|  | VPA-AR | 9 | 99.90 | 252.84 | 169.73 ± 50.78 **^b^** | 172.49 |  |
|  | VPA-AR.PRO | 9 | 114.50 | 253.14 | 174.48 ± 40.88 **^bc^** | 177.32 |  |
|  | VPA.AR-AR | 9 | 62.27 | 195.47 | 122.86 ± 47.86 **^ab^** | 124.86 |  |
|  | LPS-0 | 9 | 119.31 | 187.74 | 144.43 ± 19.26 **^ab^** | 146.77 |  |
|  | LPS-AR | 9 | 111.03 | 194.53 | 158.01 ± 29.09 **^b^** | 160.58 |  |
|  | LPS-AR.PRO.ω3 | 9 | 90.30 | 193.64 | 128.90 ± 36.37 **^ab^** | 130.99 |  |
|  | LPS.AR-AR | 9 | 85.38 | 174.97 | 136.52 ± 30.98 **^ab^** | 138.74 |  |
| GSH (µg/mg Protein) | Control | 9 | 85.30 | 108.25 | 93.42 ± 8.04 **^c^** | 100.00 | 0.001 |
|  | VPA-0 | 9 | 50.08 | 69.36 | 60.19 ± 6.08 **^a^** | 64.43 |  |
|  | VPA-AR | 9 | 66.65 | 99.35 | 86.83 ± 11.00 **^bc^** | 92.94 |  |
|  | VPA-AR.PRO | 9 | 76.56 | 109.47 | 87.94 ± 10.28 **^bc^** | 94.13 |  |
|  | VPA.AR-AR | 9 | 76.18 | 96.35 | 88.66 ± 6.55 **^c^** | 94.91 |  |
|  | LPS-0 | 9 | 65.99 | 83.62 | 74.11 ± 6.97 **^ab^** | 79.33 |  |
|  | LPS-AR | 9 | 72.42 | 111.94 | 91.40 ± 11.57 **^c^** | 97.84 |  |
|  | LPS-AR.PRO.ω3 | 9 | 82.61 | 112.58 | 92.43 ± 9.20 **^c^** | 98.93 |  |
|  | LPS.AR-AR | 9 | 82.54 | 113.60 | 99.65 ± 11.80 **^c^** | 106.67 |  |
| The groups which have different letters are significantly different with each other at significant level (0.05).  The groups which have the same letter are not significant with each other. | | | | | | | |

**Table 3.2**

| **Table 3. 2. Comparison between different groups for the intestinal leakiness parameters, One-way ANOVA test between different groups with multiple comparisons (Tukey test) within the entire groups.** | | | | | | | |
| --- | --- | --- | --- | --- | --- | --- | --- |
| **Parameters** | **Groups** | **N** | **Min.** | **Max.** | **Mean ± S.D.** | **Percent change** | **P value** |
| OCLN (ng/mg Protein) | Control | 9 | 0.10 | 0.26 | 0.19 ± 0.06 **^a^** | 100.00 | 0.004 |
|  | VPA-0 | 9 | 0.23 | 0.45 | 0.32 ± 0.08 **^bd^** | 167.76 |  |
|  | VPA-AR | 9 | 0.16 | 0.38 | 0.29 ± 0.08 **^bcd^** | 153.76 |  |
|  | VPA-AR.PRO | 9 | 0.20 | 0.35 | 0.29 ± 0.05 **^bcd^** | 151.95 |  |
|  | VPA.AR-AR | 9 | 0.21 | 0.37 | 0.25 ± 0.05 **^abc^** | 134.37 |  |
|  | LPS-0 | 9 | 0.11 | 0.36 | 0.25 ± 0.07 **^ab^** | 130.72 |  |
|  | LPS-AR | 9 | 0.20 | 0.39 | 0.29 ± 0.06 **^bc^** | 151.31 |  |
|  | LPS-AR.PRO.ω3 | 9 | 0.22 | 0.38 | 0.30 ± 0.07 **^bcd^** | 157.04 |  |
|  | LPS.AR-AR | 9 | 0.14 | 0.33 | 0.25 ± 0.06 **^ab^** | 133.88 |  |
| Zonulin (ng/mg Protein) | Control | 9 | 2.06 | 4.19 | 3.06 ± 0.81 **^a^** | 100.00 | 0.001 |
|  | VPA-0 | 9 | 10.40 | 15.62 | 12.80 ± 2.14 **^c^** | 417.59 |  |
|  | VPA-AR | 9 | 10.25 | 14.48 | 12.26 ± 1.37 **^bc^** | 400.22 |  |
|  | VPA-AR.PRO | 9 | 5.56 | 15.85 | 10.34 ± 3.07 **^bc^** | 337.56 |  |
|  | VPA.AR-AR | 9 | 7.96 | 14.66 | 10.34 ± 1.91 **^bc^** | 337.49 |  |
|  | LPS-0 | 9 | 5.19 | 16.64 | 9.93 ± 3.86 **^bc^** | 324.08 |  |
|  | LPS-AR | 9 | 5.24 | 13.39 | 9.50 ± 2.62 **^bc^** | 310.09 |  |
|  | LPS-AR.PRO.ω3 | 9 | 7.06 | 12.07 | 9.00 ± 1.63 **^b^** | 293.58 |  |
|  | LPS.AR-AR | 9 | 5.41 | 12.98 | 9.46 ± 2.12 **^bc^** | 308.76 |  |
| IFABP (ng/mg Protein) | Control | 9 | 0.03 | 0.05 | 0.041 ± 0.006 **^a^** | 100.00 | 0.001 |
|  | VPA-0 | 9 | 0.11 | 0.21 | 0.141 ± 0.030 **^d^** | 347.38 |  |
|  | VPA-AR | 9 | 0.04 | 0.13 | 0.096 ± 0.027 **^c^** | 235.47 |  |
|  | VPA-AR.PRO | 9 | 0.01 | 0.11 | 0.076 ± 0.032 **^bc^** | 186.24 |  |
|  | VPA.AR-AR | 9 | 0.02 | 0.09 | 0.044 ± 0.025 **^ab^** | 109.04 |  |
|  | LPS-0 | 9 | 0.11 | 0.21 | 0.157 ± 0.030 **^d^** | 386.59 |  |
|  | LPS-AR | 9 | 0.07 | 0.11 | 0.085 ± 0.018 **^c^** | 208.97 |  |
|  | LPS-AR.PRO.ω3 | 9 | 0.04 | 0.06 | 0.049 ± 0.005 **^ab^** | 119.55 |  |
|  | LPS.AR-AR | 9 | 0.04 | 0.05 | 0.040 ± 0.003 **^a^** | 99.16 |  |
| LBP (µg/mg Protein) | Control | 9 | 0.47 | 0.58 | 0.53 ± 0.04 **^a^** | 100.00 | 0.001 |
|  | VPA-0 | 9 | 0.83 | 1.28 | 1.00 ± 0.15 **^d^** | 190.31 |  |
|  | VPA-AR | 9 | 0.51 | 0.97 | 0.81 ± 0.14 **^bcd^** | 154.25 |  |
|  | VPA-AR.PRO | 9 | 0.25 | 0.94 | 0.72 ± 0.20 **^abc^** | 136.48 |  |
|  | VPA.AR-AR | 9 | 0.39 | 0.92 | 0.63 ± 0.17 **^abc^** | 120.14 |  |
|  | LPS-0 | 9 | 0.55 | 1.27 | 0.84 ± 0.25 **^cd^** | 158.94 |  |
|  | LPS-AR | 9 | 0.68 | 1.00 | 0.79 ± 0.12 **^bcd^** | 150.03 |  |
|  | LPS-AR.PRO.ω3 | 9 | 0.54 | 0.65 | 0.59 ± 0.03 **^ab^** | 112.88 |  |
|  | LPS.AR-AR | 9 | 0.51 | 0.64 | 0.56 ± 0.04 **^a^** | 105.87 |  |
| The groups which have different letters are significantly different with each other at significant level (0.05).  The groups which have the same letter are not significant with each other. | | | | | | | |
